# Molecular Mechanism of *Xingnao Kaiqiao* Pill for Perioperative Neurocognitive Disorder and Its Correlation With Immune and Inflammatory Signaling Pathways Based on Network Pharmacology and Molecular Docking

**DOI:** 10.3389/fnagi.2022.925072

**Published:** 2022-08-03

**Authors:** Weiwei Zhang, Gaoxiang Shi, Hui Wang, Miaomiao Feng, Xiang Gao, Qipeng Xie, Ning Zhang, Zhigan Lv

**Affiliations:** ^1^Shanxi Bethune Hospital, Tongji Shanxi Hospital, Third Hospital of Shanxi Medical University, Shanxi Academy of Medical Sciences, Taiyuan, China; ^2^Tongji Medical College, Tongji Hospital, Huazhong University of Science and Technology, Wuhan, China

**Keywords:** network pharmacology, perioperative neurocognitive disorder, drug targets, inflammatory factors, molecular docking

## Abstract

To investigate the molecular mechanism of *Xingnao Kaiqiao* Pill in the treatment of perioperative neurocognitive disorder (PND) from the perspective of network pharmacology and molecular docking technology. Active ingredients of *Xingnao Kaiqiao* Pill were screened from the traditional Chinese medicine database and analysis platform, and the putative targets were predicted. The GeneCards database was searched to obtain PND-related targets. The genes corresponding to the targets were searched and annotated on the UniProt database. The VennDiagram package in R was employed to obtain common target genes. The overlap genes were introduced into STRING to obtain a protein-protein interaction (PPI) network; thus, key targets were screened. The target relationship network of “*Xingnao Kaiqiao* Pill–traditional Chinese medicine–compound–common target” was constructed by Cytoscape software. Using R language package Bioconductor, Gene Ontology (GO) and pathway enrichment analysis (Kyoto Encyclopedia of Genes and Genomes Pathway, KEGG Pathway) were performed on the common target genes. A total of 45 active ingredients of *Xingnao Kaiqiao* Pill were screened, with 182 potential targets, and 1,579 PND-related targets were retrieved from the GeneCards databases (Score ≥ 1). Using VennDiagram, 132 overlap genes were gotten. *Xingnao Kaiqiao* Pill mainly acted on targets, such as MAPK and JUN. GO enrichment analysis displayed G protein-coupled amine receptor activity, nuclear receptor activity, ligand-activated transcription factor activity, G protein-coupled neurotransmitter receptor activity, steroid hormone receptor activity, and cytokine receptor activity. KEGG enrichment analysis exhibited 157 signaling pathways. The regulation of interleukin 17, tumor necrosis factor, hypoxia-inducible factor-1, and MAPK signaling pathways affected central nervous system (CNS) inflammatory response, cellular immunity, tumor-related signaling pathways, protected neurons, and inhibited PND. The active ingredients of *Xingnao Kaiqiao* Pill adjust interleukin 17, tumor necrosis factor, hypoxia-inducible factor-1, and MAPK signaling pathways by acting on cell targets, such as JUN, MAPK, AKT1, etc., and finally exert a therapeutic effect on PND.

## Introduction

With the increased number of elderly surgical patients, brain dysfunction after general anesthesia has been paid more and more attention in clinical practice. Neurocognitive changes related to surgery and anesthesia are mainly composed of postoperative delirium (POD) and postoperative cognitive dysfunction (POCD) (Kong et al., 2022). Since 2018, perioperative neurocognitive disorder (PND) has been used internationally to describe cognitive function changes during the perioperative period (Evered et al., 2018). PND is mainly manifested as a decline in postoperative cognitive function, which is reflected in impaired memory, attention, comprehension, and reduced ability to process information. The pathophysiological mechanism of PND is still unclear, among which neuroinflammation may be the most important mechanism (Subramaniyan and Terrando, 2019). Cytokines in the central nervous system (CNS), such as IL-6, may contribute to neuroinflammation and PND (Pereira et al., 2022). Nevertheless, there is, currently, a lack of specific therapeutic medicines.

With the clinical development of integrated traditional Chinese and western medicine, traditional Chinese medicine compound preparations have been extensively utilized in the clinical treatment and prevention of PND. For patients with PND, traditional Chinese medicine preparations *Xingnao Kaiqiao* Pill, *Angong Niuhuang* Pill, and *Buyang Huanwu* Decoction are utilized for intervention, and have certain clinical effects (Zhu and Feng, 2018). *Xingnao Kaiqiao* Pill is composed of *Gastrodiaelata Bl., Bovis Calculus, Arum Ternatum Thunb, Acoritataninowii Rhizoma, Caulis Bambusae in Taenia, Arisaematis Rhizoma, Trichosanthes Kirilowii Maxim, Coptidis Rhizoma*, and *Polygalae Radix. Xingnao Kaiqiao* Pill can calm the mind, strengthen the body resistance, eliminate pathogenic factors, suppress the hyperactive liver, relieve endogenous wind, regulate qi, resolve depression, strengthen the brain, and tonify the kidney. Modern pharmacology has found that active ingredients of above-mentioned Chinese medicines can regulate immunity, inhibit inflammation, adjust autophagy/apoptosis, and protect neurons (Hu et al., 2014; Yu et al., 2014).

With the continuous maturity of computer and database technology, the traditional Chinese medicine database and analysis platform (TCMSP) was built based on the big data of traditional Chinese medicines. The molecular network of various complex components constructed by network pharmacology and the protein/gene interactions based on multilevel targets can explain the material basis and functions of traditional Chinese medicine. Network pharmacology analysis is becoming an important method for studying complex diseases caused by multiple genes and multiple factors, such as PND. This study investigated the mechanism of *Xingnao Kaiqiao* Pill in the treatment of PND, explained the scientificity and feasibility of traditional Chinese medicine empirical medication from the perspective of molecular biology, and provided a reference for the development of targeted medicines using the TCMSP and the latest technology of Chinese medicine compound prescriptions.

## Materials and Methods

### Collection and Screening of Active Ingredients of *Xingnao Kaiqiao* Pill

The TCMSP database^1^ was employed to search the active ingredients of the *Xingnao Kaiqiao* Pill formula (*Arum Ternatum Thunb, Acoritataninowii Rhizoma, Caulis Bambusae in Taenia, Arisaematis Rhizoma, Trichosanthes Kirilowii Maxim, Coptidis Rhizoma, Bovis Calculus, Gastrodiaelata Bl*, and *Polygalae Radix*) (Ru et al., 2014). Two parameters related to ADME (absorption, distribution, metabolism, and excretion), i.e., oral bioavailability (OB) greater than or equal to 30% and drug-likeness (DL) greater than or equal to 0.18 were set to screen the obtained active ingredients.

### Investigation and Prediction of Compound-Related Targets

Chemoinformatics was employed to predict the targets of the Chinese medicine components in *Xingnao Kaiqiao* Pill using the TCMSP platform and the DrugBank database. For compounds whose targets were not found in the TCMSP, the *Pharmacopoeia* was retrieved, and literature search was conducted, and then the Pharm Mapper database was used for target prediction. The sdf format of the active ingredients was uploaded to the Pharm Mapper platform, with the property of “homo sapiens” set, and the putative targets could be obtained.

### Screening of Perioperative Neurocognitive Disorder-Related Targets

The full name of PND was entered as a search term, and the disease-related targets could be retrieved on the GeneCards database. The output results of GeneCards used a relevance score greater than 1 as the screening criteria. Considering that PND has used names, such as POD and POCD, in the past, “postoperative delirium” and “postoperative cognitive dysfunction” were also input as search terms to prevent omission; the search results were summarized, and the targets were merged.

### Annotation of Gene Names

The active ingredients of the above-mentioned medicines and the targets corresponding to the diseases were searched on the UniProt database.^2^ The UniProt ID and Gene Name were annotated for each target. The Hash function of the Perl script was employed for automatic annotation of the above targets so as to perform further analysis.

### Determination of Compound-Disease Overlap Genes

The VennDiagram package in R software was employed to calculate the overlap between the targets of *Xingnao Kaiqiao* Pill and PND, and the intersection genes corresponding to the compound disease were screened out as common target genes to determine the potential targets of *Xingnao Kaiqiao* Pill acting on PND.

### Construction of “*Xingnao Kaiqiao* Pill—Traditional Chinese Medicine—Compound—Common Target” Relationship Network

The drug names, active ingredients, and the common target genes between drug and disease were imported into the Cytoscape 3.9.1 software to construct the network diagram of “*Xingnao Kaiqiao* Pill—traditional Chinese medicine—compound—common target” relationship network for visual analysis (Shannon et al., 2003).

### Construction and Analysis of Protein-Protein Interaction Network

The above-mentioned common target genes were uploaded to the STRING 11.5 database,^3^ with the limitation of “*Homo sapiens*” as the species, and the interaction score greater than 0.9. The protein—protein interaction network was imported to the Cytoscape 3.9.1 software, and the CytoHubba plug-in was used to screen key targets encoded by hub genes. We employed four algorithms, namely, Degree, Betweenness, Closeness, and MCC to rank key targets or hub genes. The Degree value of a node shows the number of connections to that node in the network. The higher the value is, the more likely the target to be the key target.

### Gene Ontology and Kyoto Encyclopedia of Genes and Genomes Enrichment Analysis of Common Targets

GO and KEGG enrichment analyses of common target genes were performed using the ClusterProfiler package in the R software to obtain the relevant functions and pathways with false discovery rate (FDR) less than 0.05 (Wu et al., 2021). The GO enrichment categories of common target genes include biological process (BP), cellular component (CC), and molecular function (MF). The barplots, dotplots, and pathway plots were drawn by using the ggplot2 package in R, and the common target genes, namely, drug-disease intersection genes were marked on the pathway plots.

### Molecular Docking Simulation

In order to further verify the interaction between the active components of *Xingnao Kaiqiao* Pill and key targets encoded by hub genes, The RCSB PDB database (错误!超链接引用无效。was utilized to query large proteins. The structure of small molecules was obtained from the PubChem database.^4^ Potential targets and corresponding components were used to simulate molecular docking. The software programs used were AutoDock-4.2 and AutoDockTools-1.5.7 (Morris et al., 2009).

## Results

### Collection and Screening of Active Ingredients and Predictive Targets of Chinese Herbal Medicines in *Xingnao Kaiqiao* Pill

TCMSP and literature retrieval were employed to get the basic information of Chinese herbal medicines contained in *Xingnao Kaiqiao* Pill, as is shown in Table 1. The same components and targets were analyzed in Table 2. After deleting the duplicate values, 45 effective chemical components of all the single medicine in *Xingnao Kaiqiao* Pill and 182 predicted targets were determined.

**TABLE 1:** Information of ingredients in *Xingnao Kaiqiao* Pill and their putative targets.

Chinese name	Latin name	Compound number	Predict target number
Banxia	*Arum Ternatum Thunb*	12	172
Changpu	*Acoritataninowii Rhizoma*	4	103
Danxing	*Arisaematis Rhizoma*	6	81
Gualou	*Trichosanthes Kirilowii Maxim*	10	35
Huanglian	*Coptidis Rhizoma*	11	285
Niuhuang	*Bovis Calculus*	5	18
Yuanzhi	*Polygalae Radix*	1	4
			

**TABLE 2:** Single Chinese medicine with repeated targets.

Mol ID	Single Chinese medicine	Predict target number
MOL000358	*Arum Ternatum Thunb, Arisaematis Rhizoma*	38
MOL000449	*Arum Ternatum Thunb, Arisaematis Rhizoma*	31
MOL000953	*Bovis Calculus, Arisaematis Rhizoma*	4
MOL003578	*Arum Ternatum Thunb, Acoritataninowii Rhizoma*	1

### Perioperative Neurocognitive Disorder-Related Targets

The GeneCards database was searched using Relevance scores ≥ 1 as the screening criteria. The search term “perioperative neurocognitive disorders” was searched to get 124 targets, and “postoperative delirium” and “postoperative cognitive dysfunction” as the search terms to get 1,572 targets. After the results were combined and the duplicates were deleted, 1,579 PND-related targets were obtained.

### Compound-Disease Overlap Genes

**FIGURE 1:**
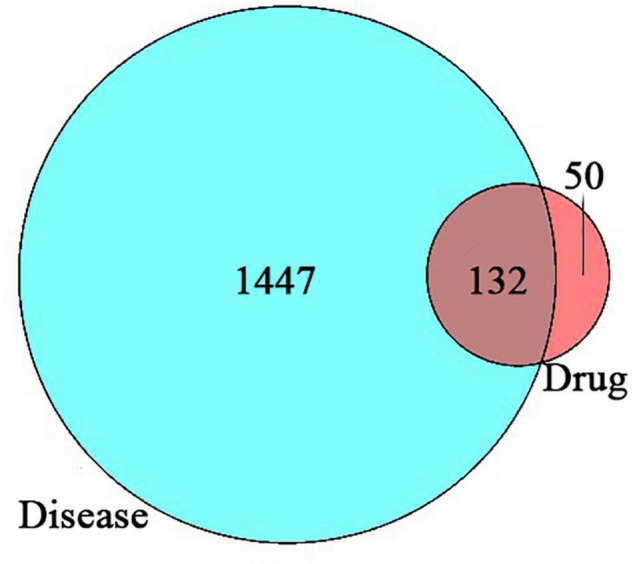
The Venn diagram of intersection targets.

Using VennDiagram, 132 overlap genes were obtained, that was, 132 potential targets of *Xingnao Kaiqiao* Pill for PND were determined, as is shown in Figure 1.

### *“Xingnao Kaiqiao* Pill—Traditional Chinese Medicine—Compound-Common Target” Relationship Network

**FIGURE 2:**
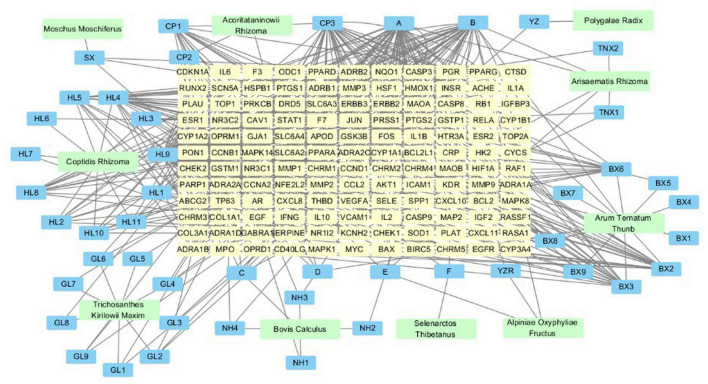
*Xingnao Kaiqiao* Pill- tradition Chinese medicine—compound-common target relationship network.

There were 234 nodes and 573 edges in the network, as is shown in Figure 2. Yellow represented the targets, blue represented the active ingredients, and green represented the medicines. The denser the connections, the more important the node is. According to the results of network analysis, stigmasterol, quercetin, beta-sitosterol, kaempferol, and baicalein determined to be the main active components of *Xingnao Kaiqiao* Pill.

### Construction and Analysis of Protein-Protein Interaction Network

**FIGURE 3:**
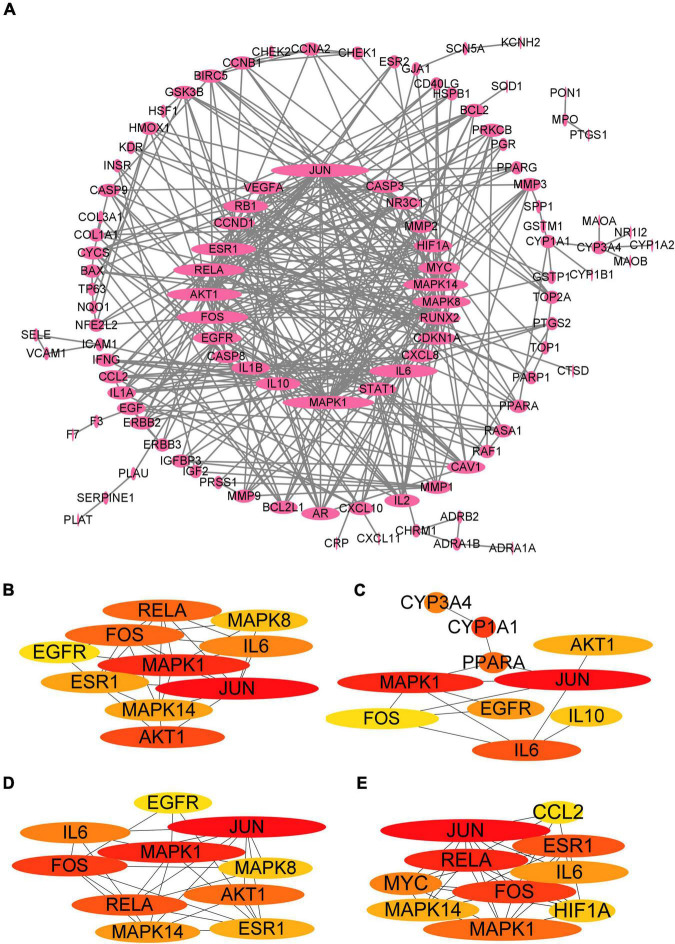
PPI network visualization and analysis. **(A)** PPI network. **(B)** The top 10 genes of Degree algorithm. **(C)** The top 10 genes of Betweeness algorithm. **(D)** The top 10 genes of Closeness algorithm. **(E)** The top 10 genes of MCC algorithm.

To shed light on the potential mechanisms through which *Xingnao Kaiqiao* Pill acts on PND, a PPI network generated from the STRING database was constructed. Cytoscape (version 3.9.1) was used to visualize the network, and the size of the nodes was proportional to the degree centrality obtained from topology analysis. As is shown in Figure 3A, the PPI network consisted of 106 non-isolated nodes and 373 edges. The top ten genes derived from the four network analysis algorithms are shown in Figures 3B–E, and the degree values of the first 10 genes are shown in Table 3. Topology analysis indicated that JUN, MAPK1, AKT1, RELA, IL6, FOS, ESR1, MAPK14, MAPK8, and EGFR were the top 10 shared targets from the perspective of degree centrality. We selected the top five targets as molecular docking targets.

**TABLE 3:** Analysis of PPI network results.

Symbol	Degree
JUN	30
MAPK1	28
AKT1	23
RELA	22
IL6	22
FOS	21
ESR1	20
MAPK14	20
MAPK8	18
EGFR	15

### Gene Ontology Enrichment Results

**FIGURE 4:**
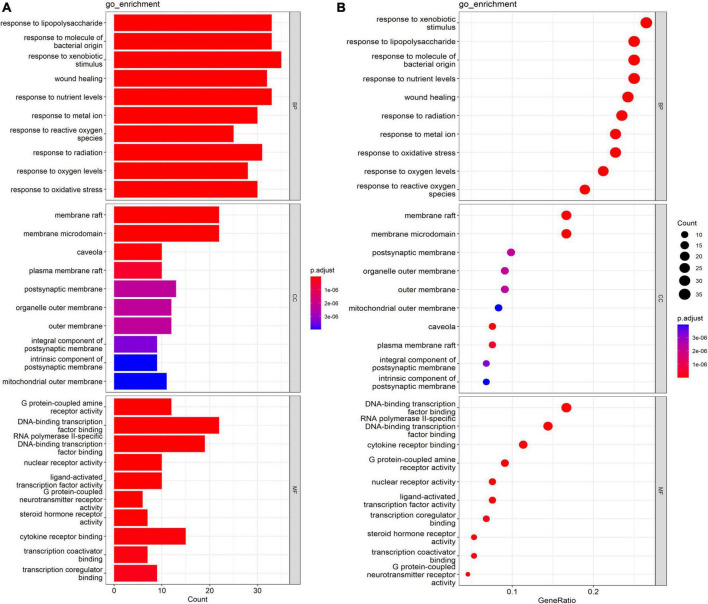
GO enrichment analysis of intersection genes. **(A)** Barplot. **(B)** Dotplot.

GO analysis results demonstrated that 132 targets were enriched in 154 GO items; these targets primarily existed in the membrane raft, the membrane microdomain, the caveola, the plasma membrane raft, the postsynaptic membrane, the organelle outer membrane, the outer membrane, the integral component of the postsynaptic membrane, and other regions of the cells. Secondly, these targets were involved in metabolism, energy pathways, apoptosis, and other biological processes. Moreover, G protein-coupled amine receptor activity, DNA-binding transcription factor binding, nuclear receptor activity, and other functions are the principal molecular functions of *Xingnao Kaiqiao* Pill against PND. The results of the top 10 in the above three aspects are shown in Figure 4.

### Kyoto Encyclopedia of Genes and Genomes Enrichment Results

**FIGURE 5:**
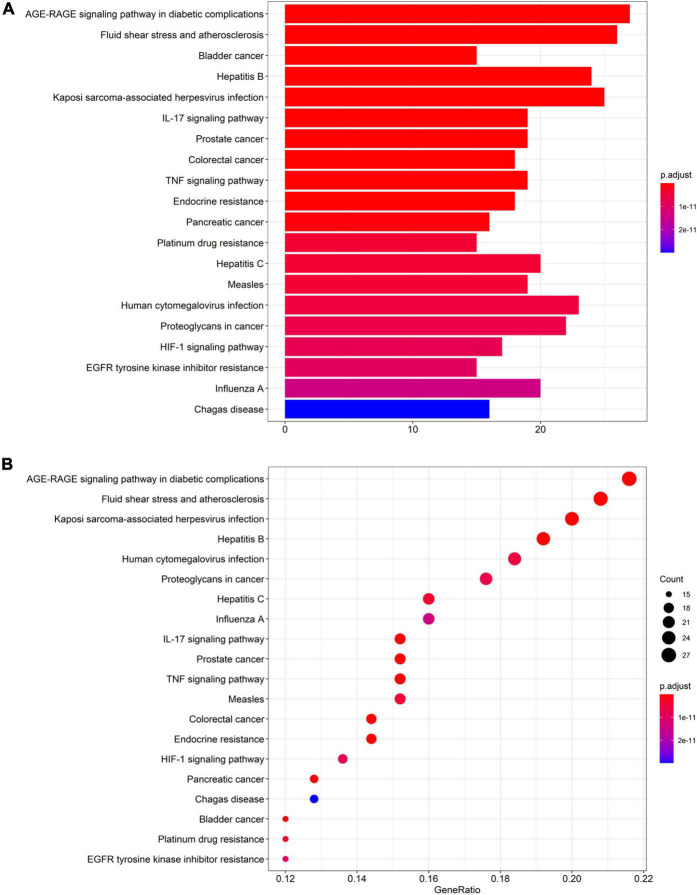
KEGG enrichment analysis of intersection genes. **(A)** Barplot. **(B)** Dotplot.

**FIGURE 6:**
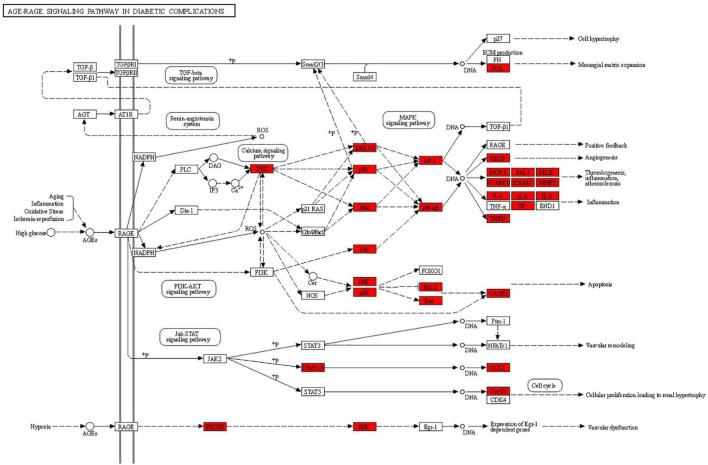
The AGE-RAGE signaling pathway.

**FIGURE 7:**
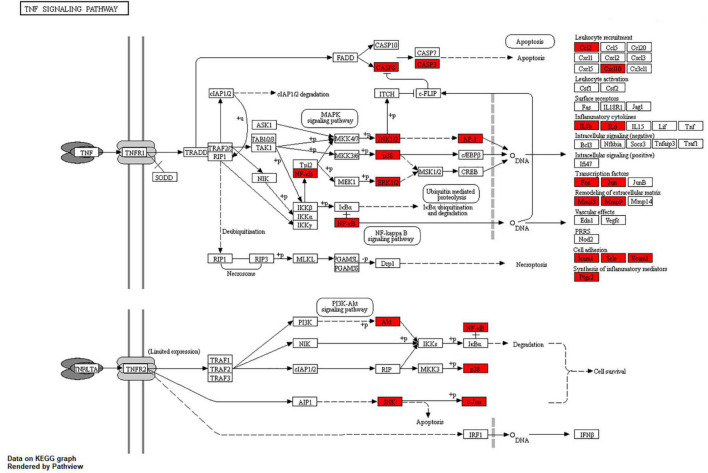
The TNF signaling pathway.

**FIGURE 8:**
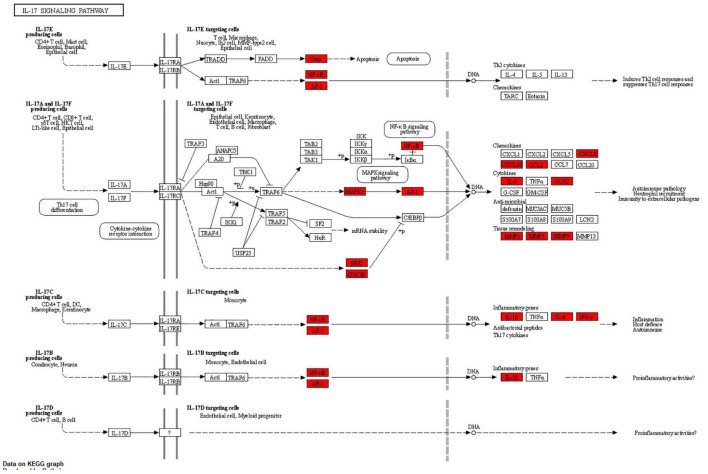
The IL-17 signaling pathway.

The results of KEGG pathway analysis of common target genes exhibited that 125 targets were enriched in 157 signaling pathways or biological processes. According to the count value and *P*-value, the first 20 items were selected, as shown in Figure 5, among which important pathways mainly involve the AGE-RAGE signaling pathway, the tumor signaling pathway, the interleukin 17 (IL-17) signaling pathway, the tumor necrosis factor (TNF) signaling pathway, the hypoxia-inducible factor-1 (HIF-1) signaling pathway, and signal pathways closely related to fluid shear stress and atherosclerosis, endocrine resistance, bladder cancer, prostate cancer, etc., which mainly play an important role in inhibiting inflammatory reaction, hormone, and endocrine regulation. The signaling pathways related to cellular immunity and inflammation consisted of the AGE-RAGE signaling pathway, the IL-17 signaling pathway, and the TNF signaling pathway; there are common targets and synergies between these signaling pathways. In conclusion, the effect of *Xingnao Kaiqiao* Pill on PND seems to have a close association with immune response and metabolism of immune molecules. The details of the above three signaling pathways are displayed from Figures 6–8, and the compound-disease intersection genes are shown in red.

### Molecular Docking Verification

**FIGURE 9:**
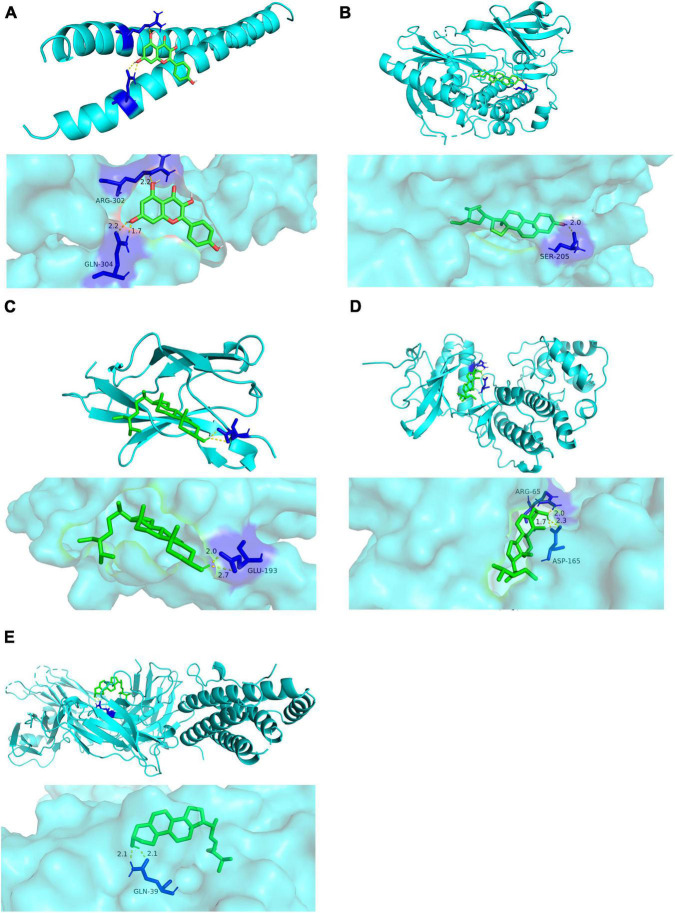
Detailed target-compound interactions with the highest molecular docking affinities. **(A)** The binding pattern between JUN and kaempferol. **(B)** The binding pattern between AKT1 and beta-sitosterol. **(C)** The binding pattern between RELA and beta-sitosterol. **(D)** The binding pattern between MAPK1 and stigmasterol. **(E)** The binding pattern between IL6 and stigmasterol.

In this study, we used molecular docking stimulation to identify the binding ability between bioactive components of *Xingnao Kaiqiao* Pill and key targets encoded by hub genes. According to Figure 9 and Table 4, all the bioactive components of *Xingnao Kaiqiao* Pill demonstrated good binding with the key targets. Beta-sitosterol had a strong binding ability with two hub genes, i.e., AKT1 and RELA. Stigmasterol showed good binding ability with MAPK1 and IL6. Kaempferol showed good binding ability with JUN.

**TABLE 4:** Docking scores of the active ingredients of *Xingnao Kaiqiao* Pill with their potential targets.

Molecular name	Affinity (kcal/mol)				
	
	JUN	MAPK1	AKT1	RELA	IL6
Stigmasterol	–4.67*	–8.44	–10.96	–7.11	–9.00
Quercetin	–3.81	–6.58	–7.49	–6.37	–6.96
Beta-sitosterol	–3.32	–7.59	–11.00	–7.18	–8.19
Kaempferol	–4.12	–6.76	–7.71	–6.50	–7.45
Baicalein	–3.16	–6.74	–6.12	–6.54	–6.78

*No hydrogen bonds formed.

## Discussion

PND, a CNS complication, presents many clinical manifestations; the deterioration of cognitive function involves cognitive, memory, motor function, attention, and concentration. According to the universally recognized pathogenesis, PND can be considered as a neuroinflammation disease (Subramaniyan and Terrando, 2019; Li et al., 2022). It has been found that there is a close connection between the nervous system and the immune system, and inhibiting the expression level of inflammatory factors or suppressing the immune response can reduce the symptoms of PND or the occurrence of PND (Mu et al., 2015).

There is no clear record of this disease in Chinese medicine. Some scholars believe that PND should be classified as “dementia,” “forgetfulness,” and “epilepsy” on the basis of its clinical manifestations (Han et al., 2011), and classified early delirium in PND as the category of “manic-depressive psychosis” in traditional Chinese medicine.

Chinese medicine treatment of PND is generally based on pathophysiological characteristics and clinical manifestations of the elderly after surgery. In a study of PND, Chinese medicine syndrome in elderly patients with bowel cancer, clinical patients were roughly divided into five syndrome types, namely, kidney essence deficiency syndrome, phlegm turbidity blocking orifice syndrome, blood stasis blocking collaterals syndrome, viscera stagnation, and turbid retention syndrome, *qi*, and blood deficiency syndrome. Elderly people frequently experience liver and kidney deficiency, insufficient *qi*, and blood. Furthermore, surgical trauma can break *qi*, consume blood, damage the collaterals, cause deficiency of *qi* and blood circulation, stagnation of blood stasis, and result in cerebral collateral blockage and mental function failure. The treatment should invigorate *qi*, activate blood, dispel blood stasis, and dredge collaterals. If the patient suffers from heart and kidney deficiency, *yin* deficiency, blood insufficiency, and internal disturbance of deficiency and fire, the treatment should nourish *yin*, blood, the heart, and focus on tranquilization. Chinese medicine compound preparation *Xingnao Kaiqiao* pill can tranquilize the emotional distress, strengthen body resistance, eliminate pathogenic factors, suppress the hyperactive liver for calming endogenous wind, regulate *qi*, dispel stagnation, tonify the kidney, and invigorate the brain. *Xingnao Kaiqiao* pill has the characteristics of overall regulation, multiple targets, multiple pathways, and multiple mechanisms, and has advantages in improving cognition and perioperative quality of life. More than 50 patients with PND, who had been consulted at the Department of Traditional Chinese Medicine in our hospital since 2020, received the treatment with *Xingnao Kaiqiao* pill and achieved good results.

Numerous research results demonstrated that PND may be induced by various factors, and its pathogenesis is not yet fully clear. Animal experiments and clinical trials have shown that PND pathogenesis is associated with increased immune inflammatory response of the CNS, abnormal neurotransmitters or receptors, abnormal cholinergic nerve function, β-amyloid formation, noxious stimulation, and stress response (Uchino et al., 2014). It is currently believed that neuroinflammatory response and excessive activation of microglia are the main factors that trigger PND in elderly patients (Subramaniyan and Terrando, 2019). Microglia are the innate immune cells of the CNS. Their excessive activation induces the release of neurotoxic substances, and then alters the normal transmission of neurotransmitters and synaptic plasticity, and ultimately leads to PND (Niraula et al., 2017). The excessive activation of microglia is strongly associated with the activation of MAPK and other inflammatory signaling pathways caused by the release of inflammatory factors in the CNS (Zhang et al., 2020).

MAPK is a class of intracellular serine/threonine protein kinases, an important signaling system that mediates extracellular signals to intracellular responses, and plays a key role in immunity, inflammation, apoptosis, and angiogenesis. The MAPK signaling pathway plays an important role in the pathophysiological process of cognitive impairment (Xiang et al., 2020). IL-6 is a multipotent cytokine with a wide range of functions; it can regulate the growth and differentiation of many kinds of cells; regulate immune response, acute phase response, and hematopoiesis; and plays an important role in immune response. Following surgery, local cytokine-driven inflammation occurs, as part of the normal healing process. Cytokines in the CNS, such as IL-6 and IL-8, may also be elevated. Perioperative changes in cytokines have a role in the development of PND (Pereira et al., 2022).

The GO analysis results confirmed that intersection genes mainly enriched in the G protein-coupled amine receptor activity, nuclear receptor activity, ligand-activated transcription factor activity, G protein-coupled neurotransmitter receptor activity, steroid hormone receptor activity, and cytokine receptor activity may be associated with abnormal neurotransmitter or receptor and abnormal cholinergic nerve function in the pathogenesis of PND (Uchino et al., 2014).

In the results of KEGG pathway enrichment, PND-related body immunity and inflammation signaling pathways include the AGE-RAGE signaling pathway, the TNF signaling pathway, and the IL-17 signaling pathway. Notably, we found the MAPK signaling pathway embedded in these pathways above. The significant indicators indicate a high correlation with the pathophysiological mechanism of PND and the pharmacological effects of *Xingnao Kaiqiao* Pill. These signaling pathways above do not exist in isolation, but have many intersections and protein interactions. The interaction of multiple signaling pathways has a key impact on the activation of downstream signaling pathways, the activation of microglia, the release of neurotoxic substances, and the impairment of cognitive function (Ding et al., 2017; Gong et al., 2020).

IL-17 is an important member of the new inflammatory cytokine family, and a proinflammatory cytokine extensively present in various inflammatory pathways (McGeachy et al., 2019). The IL-17 family is composed of six members of IL-17 (A∼F). The results of the KEGG pathway enrichment in this study exhibited that MAPK, NF-κB, GSK3β, and CASP3 sites in the compound-disease targets were crucial receptors in IL-17 target cells. Moreover, there are many types of inflammatory mediators, cytokines, and chemokines released by IL-17-mediated target cells. Among them, 10 types, such as IL-1β, IL-6, IFN-γ, etc., are visibly identified as common medicine-disease targets (Gu and Wu, 2013).

Microglia are immune cells in the CNS, and their activation pathways include three states: classic activation, alternative activation, and acquired activation. The classic activation pathway is strongly associated with TNF. The results of the KEGG pathway enrichment in this study displayed that NF-κB, JNK1/2, and Akt sites in the compound-disease target are crucial receptors in TNF target cells. There are many types of inflammatory mediators, cytokines, and chemokines released by TNF-mediated target cells. Among them, 12 types, such as IL-1β, IL-6, Jun, etc., are clearly identified as compound-disease targets.

Cellular hypoxia linked to cognitive impairment. HIF-1 is an important regulator of cells, adapting to hypoxic environment (Eltzschig and Carmeliet, 2011). HIF-1α could resist the toxic effect of Aβ, inhibits tau hyperphosphorylation, and promotes microglial activation (Schubert et al., 2009). However, the current research on the relationship between hypoxia and PND is relatively lacking.

## Conclusion

In summary, the mechanism of *Xingnao Kaiqiao* Pill in the treatment of PND is mainly associated with the regulation of CNS inflammation, and the involved signaling pathways have many intersections and protein interactions. MAPK1, JUN, AKT1, IL6, and RELA are core targets of active ingredients acting on the main related signaling pathways. Due to the limitations of the database and the algorithm, further experimental investigations are needed to verify the results from this analysis.

## Data Availability Statement

The original contributions presented in this study are included in the article/supplementary material, further inquiries can be directed to the corresponding author.

## Author Contributions

WZ and GS managed the literature searches, designed the study, interpreted the statistical analyses, and wrote the first draft of the manuscript. HW and MF contributed to the data administration. XG edited the language to improve the clarity of the manuscript. ZL instructed in the study approach and supervised the statistical analyses and their interpretation. All authors have contributed to and approved the final manuscript.

## Conflict of Interest

The authors declare that the research was conducted in the absence of any commercial or financial relationships that could be construed as a potential conflict of interest.

## Publisher’s Note

All claims expressed in this article are solely those of the authors and do not necessarily represent those of their affiliated organizations, or those of the publisher, the editors and the reviewers. Any product that may be evaluated in this article, or claim that may be made by its manufacturer, is not guaranteed or endorsed by the publisher.

## Footnotes

1
http://tcmspw.com/tcmsp.php

2
https://www.uniprot.org/

3
https://string-db.org/

4
https://pubchem.ncbi.nlm.nih.gov
